# Self-medication, self-assessment and knowledge of dental medicine students about analgesics

**DOI:** 10.4317/jced.61839

**Published:** 2024-08-01

**Authors:** Luka Šimunović, Bruno Špiljak, Krešimir Bašić, Ivana Šutej

**Affiliations:** 1 Assistant, Department of Orthodontics, School of Dental Medicine, University of Zagreb, 10000 Zagreb, Croatia; 2 PhD student, School of Dental Medicine, University of Zagreb, 10000 Zagreb, Croatia; 3 Assistant Professor, Department of Pharmacology, School of Dental Medicine, University of Zagreb, 10000 Zagreb, Croatia; 4 Associate Professor, Department of Pharmacology, School of Dental Medicine, University of Zagreb, 10000 Zagreb, Croatia

## Abstract

**Background:**

The objective of this study was to investigate the rate of self-medication among dental students and any differences and/or associations between self-medication, self-assessment and knowledge among students in later years of the same study.

**Material and Methods:**

The study was conducted between April and July 2020 at the School of Dental medicine, University of Zagreb. All students were given access to an online survey to learn more about their self-medication habits, pharmacological knowledge and analgesic self-assessment. The experimental research group consisted of 120 third- to sixth-year students (n = 120), while the control group consisted of 30 first- and second-year students who had not taken any pharmacology courses.

**Results:**

Even 110 students (91.67%) reported self-medication, with ibuprofen being the most prevalent analgesic (70%). Fifth-year students showed a statistically significant difference in all knowledge-related questions compared to third- and fourth-year students (*p*=0.0015 and *p*=0.002, respectively). In addition, in the self-assessment statements across all study years, a statistically significant difference (*p*<0.05) was also noticed. The feeling of willingness to prescribe analgesics behaved according to the increasing pattern over the years of study.

**Conclusions:**

A statistically significant difference in self-medication, self-assessment and knowledge among dental students over the years, with the alarmingly high prevalence of self-medication among them, was observed. Because effective dental practice requires a thorough knowledge of pharmaceuticals, it is vital to continually expand and refine students’ understanding of the use of analgesics in dentistry throughout their undergraduate studies.

** Key words:**Analgesics, Self Medication, Self-Assessment, Knowledge, Dentistry, Students.

## Introduction

Nonsteroidal anti-inflammatory drugs represent a group of drugs with analgesic, antipyretic and anti-inflammatory effects. Most of this group of drugs is easily available and distributed over the counter (OTC) and are high on the list of drugs most commonly used for self-medication (1). Self-medication is usually described as self-administration of medication in the absence of a current prescription with or without consultation with a healthcare professional, although there is no consensus on the definition. Self-medication extends beyond the use of OTC drugs and includes the use of prescription drugs obtained from a third party or purchased on the Internet (2). At the level of the general population, there are more and more individuals who consume a different number of drugs “on their own” regardless of development or health systematization of the countries, which is why it represents a global health challenge (2-4). The major reason for the frequent self-medication of residents of developed countries is responsibility for their health, and at the same time reducing time consuming visits to medical facilities, thinking they can solve the problem on their own (3,4). Special part of the population regarding this topic are people in the biomedical profession, including students of health sciences. Factors that contribute to this behavior among students of health sciences are the possession of adequate knowledge about medications, the level of education of the family, preference for physical activity, female gender and older age (5). At the same time, it seems that self-medication during student days can be a predisposing factor for their future professionalism and practice in this field. It could also affect the quality of providing medical care as health professionals, advising members of their families as well as patients to practice self-medication without consulting a doctor (5).

The aim of this research was to examine the frequency of self-medication among students of dental medicine, as well as to determine whether self-medication habit shows a difference and/or connection in self-assessment and knowledge among students in higher years of the same study.

## Material and Methods

-Study design

The research was conducted between April and July 2020 at the School of Dental Medicine of the University of Zagreb. Self-medication pattern, pharmacology knowledge and self assessment about analgesics were investigated through an online survey on all students attending School of dental medicine, University in Zagreb. Students from the 3rd to 6th year of study (n = 120) served as experimental research group while the control group consisted of 30 students of first and second study year which did not have attended any of pharmacology courses. The condition for joining the survey in the experimental group was taking a pharmacology course. Participation was voluntary and anonymous. The survey was distributed through closed Facebook groups for „class-related“ communication. To join the study itself, the respondents had to read and sign the informed consent, after which they were granted access to the survey itself. The survey was approved by the Ethics Committee of the School of Dental Medicine (number: 05-PA-30-XVII-5/2020) and was performed in accordance with the ethical standards of the Declaration of Helsinki.

-Questionnaire design

The questionnaire consisted of 3 parts: self-medication, knowledge and self-assessment. We assessed self-medication habit through 4 questions about the situations in which they most often take an analgesic on their own, the frequency of self-medication, and through a short answer question filled in by the respondents by entering the name of the analgesic they use most often. Knowledge was assessed through 26 questions on the mechanism of action, indications, interactions and side effects. Knowledge questions were fruther devided according to theory and practice as well as 3 stages of difficulty. Out of a total of 26 questions 16 questions were theory while 10 represented practice through the presentation of several clinical cases in which the respondents chose the one that seemed most appropriate to them from the offered therapeutic options with different analgesics. According to difficulty, we divided the questions into 8 easy (questions 1., 2., 5., 6., 7., 11., 16., 18.) 9 medium difficulty (questions 3., 4., 8., 12 .,13.,15.,19.,24.,25.) and 9 difficult questions (questions 9.,10.,14.,17.,20.,21.,22.,23.,26.). The questions regarding self-assesment were used to examine self-criticism of knowledge about the use of analgesics and they were: “I consider that I know enough about analgesics”, “I am ready to prescribe the appropriate analgesic to the patient”, “I consider that I lack knowledge about the mechanism of action”, “I consider that I lack knowledge about the dosage of analgesics “ and “I believe that I lack knowledge about the interactions of analgesics”. They were set them in the form of a Likert scale, which is a self-assessment instrument designed to measure individual perception, where for each statement the respondent could choose one of the proposed answers (1- completely agree, 2- partially agree, 3- neutral, 4- partially disagree , 5- I completely disagree) depending how statement corresponds to their self-percieved opinion.

-Statistical analyses

Descriptive statistics were processed with the Statistica program (TIBCO® Statistica™ Version 13.5.0.17., Palo Alto, CA, USA). Results were compared using one-way ANOVA test with post hoc Tukey HSD test or Kruskal-Wallis test and Mann-Whitney U test. The knowledge score was calculated using a Gaussian curve - mean value and standard deviation: X = average or good (63-74%), X + σ = very good (74-85%), X + 2σ = excellent (more than 85%) . A value below 52% resolution, i.e. less than X – σ was considered poor knowledge, while a value less than 41%, i.e. < X - 2σ was considered devastating. No control group values were used in the calculation. Correlation coefficient was tested with Pearson r test, and was considered significant at 0.05.

## Results

**Figure 1:**
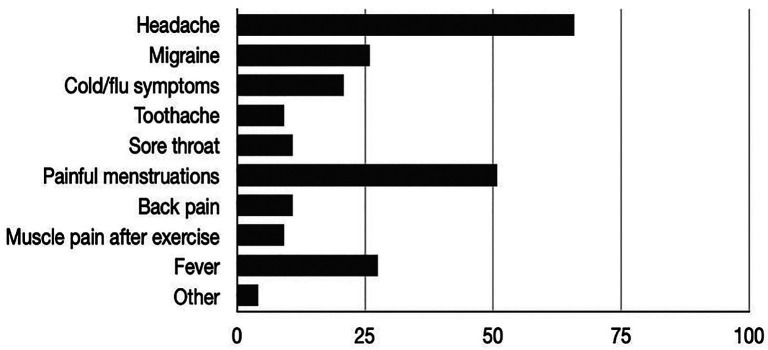
Reasons for self-medication in all subjects, presented in portion (%).

The frequency of self-medication habits among subjects are presented in  Table 1. and reasons for self medication are shown in Figure 1. Out of the total number of respondents (N=120), even 110 students (91.67%) reported self-medication, and the most commonly used analgesic was ibuprofen (70%), while paracetamol was reported by 20% of students.

 Table 2 shows the descriptive statistics of 26 questions about knowledge according to the corresponding categories and year of study, while Figure 1 shows the prevalence of reasons for self-medication in all subjects. By comparing the control group of students with all the examined years, it showed a statistically significant difference on all questions about knowledge.

A statistically significant difference in all questions about knowledge was found between students in the 5th year of study with those in the 3rd (*p*=0.0015) and 4th year of study (*p*=0.002). The difference in knowledge was statistically significant as well between students of the 3rd and 6th (*p*=0.0045) and the 5th and 6th year of study (*p*=0.0045).

In the rubric/category of theoretical questions, a statistically significant difference was observed in the knowledge of students in the 5th year of study, compared to students in the 3rd (*p*<0.001) and 4th year of study (*p*<0.001), while in clinical/practical the same questions observed between students of the 5th year of study with regard to students of the 4th (*p*=0.0125) and 6th year of study (*p*<0.001).

According to the difficulty of the questions, no statistically significant difference was found among the years of study among the easy questions. However, in the category of medium and difficult questions, the same has been proven. For moderately difficult questions, it was found between students of the 5th year of study in relation to students of the 3rd (*p*<0.001), 4th (*p*<0.001) and 6th (*p*=0.01147) years of study, while for difficult questions, the same found in students of the 3rd year of study with those of the 5th (*p*=0.011986) and 6th year of study (*p*=0.046210) as well as between students of the 4th and 5th year of study (*p*=0, 019102). The accuracy of the response to the analgesic dosage was 0.6125 +/- 0.3967. Both questions about the dosage of analgesics were answered correctly by 54 students (45%), of which 13 in the 3rd year, 11 in the 4th year, and 15 students each in the 5th and 6th.

After the questions about self-medication and knowledge, 5 questions about the self-assessment of the use of analgesics were set in the form of a Likert scale in which answers from 1 to 5 were suggested (1- completely agree, 2- partially agree, 3- neutral, 4- partially disagree, 5- completely disagree).

The distribution of responses to statements between all years of the study is statistically significant (*p* < 0.05).

On the first statement, “I consider that I lack knowledge about the mechanism of action”, the most significant fact is that none of the third year students completely agrees with the statement.

On the statement “I consider that I lack knowledge about the dosage of analgesics”, there is a trend of absolute agreement with the statement, meaning that the higher year of study, has a smaller proportion of the students who absolutely agree that they lack knowledge about dosage.

Among third, fourth and fifth year of study, the proportion of students who partially agree is higher while this trend is not present among the sixth year of study on the statement “I believe that I lack knowledge about the interactions of analgesics”.

For the statement “I consider that I know enough about analgesics”, the most important fact is that no one completely agrees with the statement. As well, a bigger portion of fifth year students, rather than sixth year, partially agrees with the claim.

**Figure 2:**
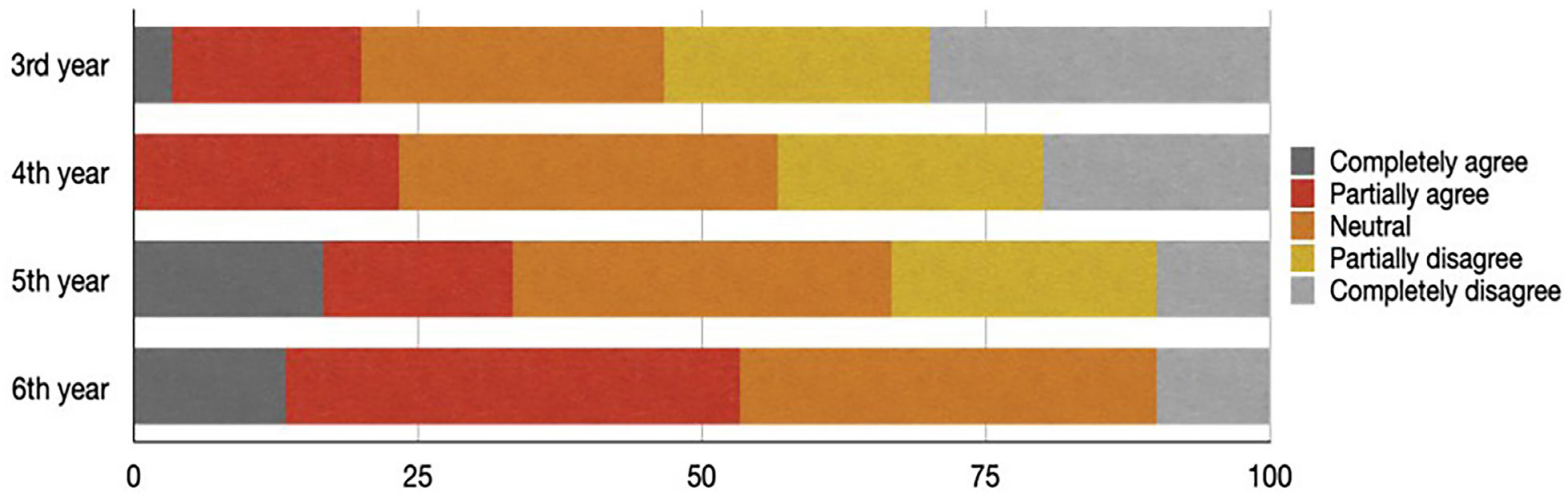
Distribution (%) of answers on the question “I feel ready to prescribe an appropriate analgesic to the patient”.

The most interesting fact of the last statement of the self-assessment “I feel ready to prescribe an appropriate analgesic to the patient” is that none of the fourth-year students feel absolutely ready to prescribe analgesics to the patient (Fig. 2).

Pearson’s test found a statistically significant correlation between the accuracy of the survey and the self-assessment of willingness to prescribe analgesics (r 0.1844), as well as between the frequency of self-medication and the willingness to self-prescribe analgesics (r 0.2205). The feeling of readiness to prescribe analgesics behaves according to the pattern of increasing over the years of study (3rd year the least ready, while 6th year the most ready).

The higher the year of study (*p*<0.05).

1. They have more correct answers to all questions (r 0.2115) 

2. They self-medicate more often (r 0.2102) 

3. They believe that they lack knowledge about the mechanism of action ( r 0.1966) 

4. They believe that they have sufficient knowledge about dosage (r 0.2683) 

5. They feel more ready to prescribe analgesics independently (r 0.3421)

## Discussion

Pharmacology plays an important role in dentistry. Knowledge of pharmacokinetics and pharmacodynamics of a given drug is necessary in order to successfully treat a dental condition using the drug. In the curriculum of the School of Dental Medicine in Zagreb, the subject of pharmacology is held in the 3rd year, and clinical pharmacology in the 5th year of study.

The subjects of this study reported self-medication pattern most commonly as 2-3 times a month (3rd year less than 5x a year, 4th and 5th year 2-3x a month and 6th year more than 5x a year, but less than 2-3x a month). Out of the total number of respondents, 110 students (91.67%) self-medicate, which is almost identical (in an interval of up to 5% difference) to the results of research conducted in Iraq (6) and France (7). Approximately similar results (range 5-20% difference) were shown by studies conducted in Bangladesh (8), Saudi Arabia (9,10), India (11,12), Nepal (13), Serbia (5) and Poland (14), while this is not the case with the results of studies conducted in Ethiopia (15), Nigeria (16), Saudi Arabia (17,18), Egypt (19), Nepal (20) and China (21) in which the percentage of self-medication ranges from 49.3 to 65% (interval >20% difference). We find the explanation of such results in the easy and simple availability of this class of medicine and the strong presence of traditional medicine in certain countries of the world, however, future research is needed to confirm exact reason.  Table 3 shows the results of self-medication of students in previously conducted research (not older than 10 years), found in the available literature.

The most common reason for self-medication in our study was headache followed by painful menstruations and fever (Fig. 1). A number of studies also mention headache as the most common cause (9,15-18,20) which corresponds with our results. It is interesting to emphasize that there was a correlation between the willingness to prescribe analgesics and self-medication. Higher year of study correlated with more frequent use of analgesics “on their own”, which is in accordance with the results of the research by Tesfaye *et al*. (22), but in contrast to the research of Shrestha *et al*. (20) in which students of the first semester of medical and dental studies had a greater tendency to self-medicate. The explanation for this correlation can be that older students showed greater knowledge about pharmacology of analgesic medication, therefore know more about them and probably for this reason believe that they are able to estimate when and how often to self-medicate with them. This should be investigated further through qualitative research. Ibuprofen as the most commonly used analgesic in Croatia was also confirmed in our previous studies (23,24), as well as in the study by Guzman-Alvarez *et al*. conducted in Mexico (25). Diclofenac was the most commonly used in the study by Jain *et al*. (26) while the same applies to paracetamol in a series of studies carried out so far (7,9,11,14,18). The variety of analgesics in self-medication in different countries could be explained by the study of Ryvak and Denysiuk from 2019 (27), who found that advertising influences the choice of a drug in the treatment of more than a quarter of respondents in the general population. Although most believe that the marketing of pharmaceutical companies does not influence their decision, almost two-thirds of them (62.8%) buy drugs from pharmacies that they heard about through advertising for self-medication. A great precaution should be taken regarding analgesic use, since paracetamol has been proven to be the most common drug for self-poisoning, and accordingly certain countries have introduced restrictions on its prescription (28). The best knowledge was determined by this survey in the 5th year of study. The explanation for the better knowledge in the 5th than in the 6th year of study is found in the fact that students in the 5th year of study have the opportunity to attend the clinical pharmacology course, which additionally prepares them for future clinical work. The same pattern was found in the knowledge difference between the 3rd and 4th year in relation to the subject of pharmacology. Consequently, we believe that the course material is clinical pharmacology, i.e. pharmacology at the time of completing the survey was more recent for the 5th and 3rd year students than for the 6th and 4th year students. The results of the control group, where the average for all questions is 38.21%, show the importance of both courses - pharmacology in the 3rd year and clinical pharmacology in the 5th year of study (Table 1). The dosing knowledge of our respondents (45%) correlates with the dosing knowledge of the study by Jain *et al*. conducted in 2016 (26). Given that this is less than half of the total number of respondents, we consider this result unsatisfactory, which also suggests to us that it is necessary to invest even greater efforts in transferring knowledge to students about the mentioned topic. As part of the pharmacology curriculum, students are taught the importance of rational drug use. Health care students, with the gained knowledge, should be aware of the consequences that irrational medication use can bring. Given their future role in patient care and education, it is important to understand students’ practices and self-beliefs regarding self-medication. However, earlier studies reported that the healthcare student population is also unrational in practicing self-medication (29,30). Self-medication practice among healthcare students and workers poses a serious threat to professionalism and biomedicine and potentially jeopardizes public confidence in the profession. To prevent the escalation of this problem a holistic approach is preferable and also needed. Such an approach includes increased awareness and education about the advantages and disadvantages of self-medication, strategies to prevent the supply of over-the-counter drugs to pharmacies, and stronger legislation to control drug advertising (5). Additionally, student health-promoting groups at universities could raise awareness of these issues among their friends with the help of pharmacists or doctors. The creation of multimedia programs or animations could also be helpful in improving the knowledge of the general population about the potential dangers of self-medication (21). Our study has several limitations. Since this is a cross-sectional study describing the current situation, each variable was measured only once, and exposure and outcome were assessed simultaneously. For this reason, we cannot interpret evidence of any association before causation can be established. The next limitation is the absence of comparative groups, such as students from other universities in the country. Also, although students are encouraged to fill out the questionnaire independently, mutual influences among respondents cannot be completely excluded.

However, despite all this, this study includes a representative sample of the dental student population as the School of Dental Medicine in Zagreb is the largest dental faculty in the Republic of Croatia.

Taking into account all of the above, future research should still be conducted on an even larger sample of dental medicine students in order to determine whether the results of this study are representative for all dental medicine students in Croatia, as well as at the international level.

## Conclusions

This study showed a statistically significant difference in self-medication, self-assessment, and knowledge between the years among students of dental medicine, and that better results in knowledge are related to the actual pharmacology courses taken. Self-medication practice correlated with the higher knowledge and the higher years of study. In addition, the prevalence of self-medication with analgesics is alarmingly high among students of dental medicine in the city of Zagreb. Thorough knowledge of drugs is necessary for good dental practice, and therefore it is important to expand and iterate students’ knowledge about the use of analgesics in dental medicine throughout the study.

## Figures and Tables

**Table 1:** Frequency of self-administration of analgesics according to the year of study.

Frequency	3rd year	4th year	5th year	6th year	Total
2-3 times a week	0 (0%)	0(0%)	3(10%)	5(16,67%)	8 (6,67%)
Once a week	3(10%)	2(6,67%)	4(13,33%)	2(6,67)	11 (9,17%)
2-3 times a month	6(20%)	12(40%)	15(50%)	7 (23,33%)	40 (33,33%)
More than 5 times a year	8(26,67%)	10(30%)	2(6,67%)	9 (30%)	29 (24,17%)
Less than 5 times a year	11 (36,67%)	6(20%)	2(6,67%)	5 (16,66%)	24 (20%)
Never	2 (6,67%)	0(0%)	4(13,33%)	2 (6,67%)	8 (6,67%)

**Table 2:** Descriptive statistics of the accuracy of answers to questions about knowledge by year of study (N=30 per year). * - statistically significant difference (*p*<0.05), SD - standard deviation.

	3rd year		4th year		5th year		6th year		Control	
	Mean	SD	Mean	SD	Mean	SD	Mean	SD	Mean	SD
All questions	0,6026	0,0905*	0,5833	0,1093	0,7013	0,0983*	0,6321	0,1058	0,3821	0,1281
Theory	0,6176	0,0843*	0,6039	0,1398	0,7118	0,0662*	0,6392	0,0856*	0,3961	0,1301
Practice	0,5741	0,2086	0,5444	0,1581	0,6815	0,2058	0,6185	0,2383	0,3556	0,1877
Difficult questions	0,3593	0,1743	0,3741	0,1663	0,4889	0,1836	0,4444	0,1459	0,2148	0,1675
Medium questions	0,5852	0,1542	0,5481	0,1773	0,7404	0,1283	0,6185	0,1791	0,3815	0,1994
Easy questions	0,8958	0,1042	0,8583	0,1382	0,8958	0,0933*	0,8583	0,1217	0,5708	0,2170

**Table 3:** Results on self-medication of students in previously conducted studies.

Author (year) Journal (reference)	Respondents (N)	Country	Self-medication of students (%)
Akande-Sholabi et al. (2021) J Pharm Policy Pract (16)	866 students	Nigeria	54.6
Al-Ameri et al. (2017)East Mediterr Health J (6)	1435 students	Iraq	92.4
Al-Qahtani et al. (2022)Risk Manag Healthc Policy (17)	205 students	Saudi Arabia	60.0
Alam et al. (2015) BMC Res Notes (8)	500 students	Bangladesh	100.0
Albusalih et al. (2017)Pharmacy (Basel) (18)	450 students	Saudi Arabia	49.3
Al Essa et al. (2019)Saudi Pharm J (9)	272 students	Saudi Arabia	73.2
Chindhalore et al. (2020)J Educ Health Promot (11)	216 students	India	83.3
Gras et al. (2020)Therapie (7)	1257 students	France	95.0
Gyawali et al. (2015)J Clin Diagn Res (13)	295 students	Nepal	81.9
Helal, Abou-ElWafa et al. (2017)Int J Environ Res Public Health (19)	800 students	Egypt	62.9
Ibrahim et al. (2015)Pakistan J Med Sci (10)	504 students	Saudi Arabia	75.2
Kasulkar Gupta et al. (2015)Indian J Pharm Sci (12)	488 students	India	71.9
Lukovic et al. (2014)PLoS One (5)	1296 students	Serbia	79.9
Shrestha et al. (2021)JNMA J Nepal Med Assoc (20)	199 students	Nepal	50.3
Tse et al. (2017)Cyberpsychol Behav Soc Netw (21)	387 students	Hong Kong, China	55.3
Wiliński et al. (2015)Folia Med Cracov (14)	250 students	Poland	98.8
Zewdie et al. (2020)Risk Manag Healthc Policy (15)	341 student	Ethiopia	65.0

## Data Availability

The datasets used and/or analyzed during the current study are available from the corresponding author.
